# Contraceptive Use and Uptake of HIV-Testing among Sub-Saharan African Women

**DOI:** 10.1371/journal.pone.0154213

**Published:** 2016-04-25

**Authors:** Katherine E. Center, Jayleen K. L. Gunn, Ibitola O. Asaolu, Steven J. Gibson, John E. Ehiri

**Affiliations:** 1 Department of Obstetrics and Gynecology, University of Arizona, Tucson, Arizona, United States of America; 2 Biomedical Research and Education Foundation of Southern Arizona, Tucson, Arizona, United States of America; 3 Department of Epidemiology and Biostatistics, Mel and Enid Zuckerman College of Public Health, University of Arizona, Tucson, Arizona, United States of America; 4 Department of Health Promotion Sciences, Mel and Enid Zuckerman College of Public Health, University of Arizona, Tucson, Arizona, United States of America; 5 University of Arizona Cancer Center, Tucson, Arizona, United States of America; University of Pittsburgh, UNITED STATES

## Abstract

Despite improved availability of simple, relatively inexpensive, and highly effective antiretroviral treatment for HIV/AIDS, the disease remains a major public health challenge for women in sub-Saharan Africa (SSA). Given the numerous barriers in access to care for women in this region, every health issue that brings them into contact with the health system should be optimized as an opportunity to integrate HIV/AIDS prevention. Because most non-condom forms of modern contraception require a clinical appointment for use, contraception appointments could provide a confidential opportunity for access to HIV counseling, testing, and referral to care. This study sought to investigate the relationship between contraceptive methods and HIV testing among women in SSA. Data from the Demographic and Health Survey from four African countries—Congo, Mozambique, Nigeria, and Uganda—was used to examine whether modern (e.g., pills, condom) or traditional (e.g., periodic abstinence, withdrawal) forms of contraception were associated with uptake of HIV testing. Data for the current analyses were restricted to 35,748 women with complete information on the variables of interest. Chi-square tests and logistic regression models were used to assess the relationship between uptake of HIV testing and respondents' baseline characteristics and contraceptive methods. In the total sample and in Mozambique, women who used modern forms of contraception were more likely to be tested for HIV compared to those who did not use contraception. This positive association was not demonstrated in Congo, Nigeria, or Uganda. That many women who access modern contraception are not tested for HIV in high HIV burden areas highlights a missed opportunity to deliver an important intervention to promote maternal and child health. Given the increasing popularity of hormonal contraception methods in low-income countries, there is an urgent need to integrate HIV counseling, testing, and treatment into family planning programs. Women on hormonal contraceptives should be encouraged to continue to use condoms for HIV-prevention.

## Introduction

In 2013, 35 million people worldwide were living with HIV, of which 2.1 million had become newly infected [1]. Roughly 70% of the world’s HIV positive individuals—25 million people—reside in sub-Saharan Africa (SSA) [1,2]. The epidemic has had widespread effects, including putting increased pressure on healthcare facilities to diagnose and treat HIV. Women in SSA are disproportionately infected with HIV via heterosexual contact during their reproductive years [2].

Finding ways to mitigate the HIV epidemic has long been a goal of researchers and world leaders alike. Abstinence and condom use are two of the three endorsed behavioral strategies recommended by HIV prevention programs, emphasizing the ABC approach to HIV reduction—i.e. abstinence, be faithful, and condom use programs. There is little doubt that condom use and abstinence provide proven prevention options in reducing HIV transmission [3,4]; however, it has become increasingly apparent that the ABC approach over simplifies the complex nature of the HIV epidemic [5–7].

Although SSA has one of the lowest rates of contraceptive use globally [8], the use of modern forms of contraception (e.g., pills, IUD, condom, diaphragm) in this region has changed only minimally from 2008–2014 (23.6% vs. 27.6%) [9, 10]. An estimated 19.7% of married or unionized women use modern contraceptive methods compared to 5.4% who use traditional methods [8]. Currently, injectable contraception is the most prevalent form of contraception used across SSA [11,12].

Apart from condoms, no other form of contraception has been found to protect against sexually transmitted infections, including HIV. Both the World Health Organization (WHO) and the United States Agency for International Development (USAID) have acknowledged the unclear relationship between the use of hormonal contraceptives and HIV acquisition [13]. Not only are non-condom contraceptives ineffective in protecting against sexually transmitted infections in general, but hormonal contraceptives specifically have also been implicated in higher rates of HIV infection. In Asia, female sex workers were more likely to be HIV positive if they took oral contraceptives (OC) compared to female sex workers who did not take OC [13–15]. A meta-analysis exploring the relationship OC among African women and HIV infection found a 45% increase in seroprevalent or seroincident HIV cases among women who were using oral contraceptives (OR 1.45: 95% CI 1.15–1.83) [16]. In contrast, a different meta-analysis in SSA did not find OC to be related to risk in HIV infection [17]. Thus, the WHO and USAID advocate the use of dual contraception, which includes using both a highly effective contraception—e.g. an injectable hormonal contraception—to help prevent pregnancy and condoms to prevent the transmission of sexually transmitted infection [13,15]. However, fear of unintended pregnancy—not necessarily fear of HIV—promotes condom use [18].

Because more women in SSA are using non-condom methods of contraception, it is even more important that they understand the importance of HIV testing [2,19]. Thus, it is essential to understand the effects that contraceptive use has on the uptake of HIV testing in high HIV transmission areas of SSA. The objective achieved in this study was to examine how modern and traditional forms of contraception were associated with uptake of HIV testing. The knowledge gained from this study can aid policy makers in targeting HIV education and testing to women in SSA.

## Methods

### Survey

Data was derived from the Demographic and Health Survey (DHS) by combining information from four unique African countries: Congo (Brazzaville, 2011–2012), Mozambique (2011), Nigeria (2013), and Uganda (2011). The DHS is a nationally representative cross-sectional survey. Each country was chosen to represent a geographical location across the continent with Congo (Brazzaville) representing central Africa (DHS 2011–2012), Mozambique representing southern Africa (DHS 2011), Nigeria representing western Africa (DHS 2013), and Uganda representing eastern Africa (DHS 2011). Detailed information regarding the characteristics and collection of the DHS data have been previously published [20]. Data for the current analyses were restricted to women with complete information on the variables of interest.

### Measures

Comprehensive knowledge of HIV was created using UNICEF’s definition [21]. Women were classified as having comprehensive HIV knowledge if they correctly: 1) acknowledged that a healthy-looking person can have HIV; 2) identified two methods of preventing sexual transmission of HIV; and 3) rejected two common misconceptions about HIV transmission—HIV can be contracted from mosquito bites; or HIV can be contracted by sharing food with someone living with AIDS. Respondents’ religion was classified into the following categories: Catholic, Muslim, Christian—other (any denomination excluding Catholicism), and Other/No religion. For these analyses, contraception was classified as either: modern contraception which describes hormonal (pills, injectables, IUDs, and implants), sterilization (both female and male), or barrier contraception (condom or diaphragm); or traditional which includes periodic abstinence, withdrawal, and other country specific forms of contraception. HIV stigma was measured as respondents answering ‘No’ to any of the following items: Willing to care for a relative with HIV; A female teacher infected with HIV, but is not sick, should continue teaching; I would buy vegetables from a vendor with HIV [22].

### Statistical Analysis

For our analysis, only individual DHS data was used. Due to oversampling of certain populations, individual weights were used as recommended by DHS to adjust for nonresponse to questions and to make the data more representative on a national level [23]. Chi-square tests and logistic regression models were used to assess the relationship between uptake of HIV testing and respondents’ baseline characteristics and contraceptive methods. Because condoms are the only form of contraception known to decrease the spread of HIV, a sensitivity analysis was conducted by reanalyzing the relationship between HIV testing and contraceptive use in both the crude and adjusted logistic regression models, excluding women who reported using condoms. Statistical significance was set at p< 0.01 for all models, and data analyses were done using the ‘PROC SURVEY’ command. All data cleaning and analyses were conducted using SAS 9.4 (Cary, North Carolina).

DHS surveys were conducted under the scientific and administrative oversight of the local country, including ethical review by the corresponding local ethics review board. Data collection procedures were also approved by the ORC Macro institutional review board. In addition, this secondary data-analysis was evaluated by the Mel and Enid Zuckerman College of Public Health Research Office at the University of Arizona, and was considered exempt from human subjects review.

## Results

Of the 35,748 women included in the analyses, 15.7% were Congolese, 37.5% were Mozambicans, 22.6% were Nigerians, and 24.2% were Ugandans.

Table 1 shows the weighted results of the participant characteristics at an aggregate level and by country. In the pooled and individual country analysis, women aged 20–29 represented the largest proportion of women sampled (37.1% overall). Sociodemographic and economic characteristics varied between countries. Congo had the largest proportion of women with a secondary education (67.5%), while Mozambique had the largest proportion of women with no formal education (30.6%). The largest proportion of women living in rural areas was found in Uganda (80.2%). Mozambique was the only country where over half of women reported they had difficulty accessing a healthcare facility (52.5%). Although the DHS is a representative survey, the final sample of women in this study were in the richest quintile of the wealth index. Nigeria had the largest proportion of women who were currently married (65.5%). More women in Uganda had ≥8 children (12.6%) than any other country. The most common religion in each country was Christian (non-Catholic) (49.1% overall). Nigeria was the only country with over half of the sample endorsing stigmatizing women with HIV (58.9%). HIV knowledge across all countries was under 50% (Congo, 41.8%; Mozambique, 31.7%; Nigeria, 45.5%; and Uganda, 37.9%). Mozambique and Uganda both had high proportions of women who were not utilizing any form of contraception (87.7% and 76.4%, respectively). In Congo, high proportions of women reported using traditional methods of contraception (41.8%). Of the women who used modern forms of contraception, most (N = 4,668) used hormonal or barrier (N = 4,169) methods, with few using IUD (N = 360) or sterilization (N = 329) (results not shown). HIV testing was highest in Uganda (75.6%) followed by Nigeria (58.4%) and Mozambique (50.4%). Congo was the only country where under half of women (44.0%) were tested for HIV.

**Table 1:** Weighted characteristics of participants.

	Total		Congo		Mozambique		Nigeria		Uganda	
	N	%	N	%	N	%	N	%	N	%
**Age**										
15–19	6545	18.3	893	15.9	2974	22.2	646	8.0	2032	23.5
20–29	13272	37.1	2380	42.3	4633	34.5	3070	38.0	3190	36.9
30–39	10097	28.3	1704	30.3	3595	26.8	2693	33.4	2105	24.4
40–49	5833	16.3	644	11.5	2211	16.5	1665	20.6	1313	15.2
**Education**										
None	6155	17.2	200	3.6	4104	30.6	735	9.1	1116	12.9
Primary	14634	41.0	1214	21.6	6776	50.5	1518	18.8	5126	59.3
Secondary	12127	33.9	3796	67.5	2347	17.5	4038	50.0	1946	22.5
Tertiary	2831	7.9	411	7.3	185	1.4	1783	22.1	453	5.2
**Residence**										
Urban	15541	43.5	3945	70.2	4719	35.2	5164	64.0	1712	19.8
Rural	20207	56.5	1676	29.8	8693	64.8	2909	36.0	6928	80.2
**Difficulty accessing healthcare facility**										
No	21654	60.6	3451	61.5	6367	47.5	6776	84.2	5059	58.6
Yes	14059	39.4	2157	38.5	7045	52.5	1276	15.8	3581	41.4
**Wealth Index**										
Poorest	5076	14.2	793	14.1	2529	18.9	241	3.0	1513	17.5
Poorer	5719	16.0	1065	18.9	2457	18.3	625	7.7	1572	18.2
Middle	6517	18.2	1158	20.6	2479	18.5	1282	15.9	1597	18.5
Richer	8045	22.5	1308	23.3	2740	20.4	2276	28.2	1722	19.9
Richest	10391	29.1	1298	23.1	3208	23.9	3649	45.2	2237	28.9
**Marital Status**										
Never in a union	8033	22.5	1398	24.9	2443	18.2	2089	25.9	2103	24.3
Married	14995	41.9	602	10.7	6024	44.9	5286	65.5	3083	35.7
Living with partner	8492	23.8	2746	48.8	3107	23.2	311	3.8	2328	27.0
Widowed/Divorced	4227	11.8	876	15.6	1839	13.7	387	4.8	1127	13.0
**Gravidity**										
None	8314	23.3	1132	20.1	2886	21.5	2093	25.9	2203	25.5
1 to 3	13961	39.0	2895	51.5	5678	42.3	2697	33.4	2692	31.2
4 to 7	10932	30.6	1443	25.7	4028	30.0	2809	34.8	2653	30.7
More than 8	2540	7.1	152	2.7	821	6.1	474	5.9	1092	12.6
**Religion**										
Christian-Catholic	10679	29.9	1846	32.8	3892	29.0	1431	17.7	3509	40.6
Muslim	5419	15.2	33	0.6	2288	17.1	1977	24.5	1121	13.0
Christian-Other	17556	49.1	3353	59.7	5676	42.3	4619	57.2	3907	45.2
Other Religion	2094	5.8	388	6.9	1557	11.6	46	0.6	103	1.2
**HIV Stigma**										
No	19346	54.1	2876	51.2	8220	61.3	3320	41.1	4930	57.1
Yes	16402	45.9	2745	48.8	5193	38.7	4753	58.9	3710	42.9
**HIV knowledge**										
No	22192	62.1	3272	58.2	9156	68.3	4397	54.5	5367	62.1
Yes	13556	37.9	2349	41.8	4257	31.7	3677	45.5	3273	37.9
**Contraception**										
None	21859	61.1	1116	19.9	11760	87.7	2377	29.4	6606	76.4
Modern	9434	26.4	2154	38.3	1619	12.1	3881	48.1	1780	20.6
Traditional	4455	12.5	2351	41.8	34	0.2	1816	22.5	255	3.0
**HIV Testing**										
Yes	20473	57.3	2472	44.0	6754	50.4	4716	58.4	6531	75.6
No	15275	42.7	3148	56.0	6659	49.6	3358	41.6	2110	24.4

Note: Each N and percentage represents a weighted number calculated by using the DHS weighted sampling unit and PROC SURVEY’ command in SAS.

Table 2 shows the relationship between uptake of HIV testing and respondents’ baseline characteristics and contraceptive methods. All Chi-square tests regarding the relationship between HIV testing and participants’ sociodemographic, HIV knowledge or contraceptive use were statistically significant (p<0.01) with the exceptions of difficulty accessing a healthcare facility in Congo (p = 0.07) and Uganda (p = 0.91); religion in Uganda (p = 0.58); and contraception use in Nigeria (p = 0.47).

**Table 2:** Chi square testing the relationship between uptake of HIV testing and participant characteristics.

	Total			Congo			Mozambique			Nigeria			Uganda		
	Not Tested %	Tested %	*p*	Not Tested%	Tested %	*p*	Not Tested%	Tested %	*p*	Not Tested %	Tested %	*p*	Not Tested%	Tested%	*p*
**Age**															
15–19	65.8	34.2	< .01	73.9	26.1	< .01	71.6	28.4	< .01	75.5	24.5	< .01	50.7	49.3	< .01
20–29	32.9	67.1		51.8	48.1		33.6	66.4		38.9	61.1		12.0	88.0	
30–39	35.4	64.6		50.0	50.0		41.9	58.1		32.4	67.6		16.1	83.9	
40–49	52.0	48.0		62.5	37.5		66.3	33.7		48.2	51.7		27.2	72.8	
**Education**															
None	55.4	44.5	< .01	73.9	26.1	< .01	58.6	41.4	< .01	72.6	27.4	< .01	29.3	70.7	< .01
Primary	43.7	56.2		69.2	30.8		50.7	49.3		52.8	47.2		25.9	74.1	
Secondary	40.9	59.1		54.7	45.3		33.8	66.2		41.7	58.3		20.9	79.1	
Tertiary	17.4	82.6		21.0	79.0		12.6	87.4		18.9	81.1		10.2	89.8	
**Residence**															
Urban	38.4	61.6	< .01	50.3	49.7	< .01	38.2	61.8	< .01	36.1	63.9	< .01	18.2	81.8	< .01
Rural	46.1	53.9		69.5	30.5		55.8	44.2		51.3	48.7		25.9	74.1	
**Difficulty accessing healthcare facility**															
No	38.6	61.4	< .01	54.3	45.7	0.07	40.5	59.5	< .01	39.5	60.5	< .01	24.5	75.5	0.91
Yes	49.0	51.0		58.6	41.4		57.9	42.1		52.3	47.7		24.3	75.7	
**Wealth Index**															
Poorest	55.8	44.2	< .01	78.3	21.7	< .01	65.6	34.4	< .01	74.6	25.4	< .01	24.6	75.4	< .01
Poorer	51.9	48.1		64.4	35.6		61.3	38.7		58.2	41.8		26.4	73.6	
Middle	48.3	51.7		57.4	42.6		54.6	45.4		55.5	44.5		26.3	73.7	
Richer	40.9	59.1		49.1	50.9		40.4	59.6		46.5	53.5		27.9	72.1	
Richest	29.2	70.8		41.3	58.7		32.2	67.8		28.6	71.4		18.9	81.1	
**Marital status**															
Never in a union	59.6	40.4	< .01	66.9	33.1	< .01	67.0	33.0	< .01	54.7	45.3	< .01	50.7	49.3	< .01
Married	36.9	63.1		49.8	50.2		46.9	53.1		36.3	63.7		15.8	84.2	
Living with partner	38.2	61.8		52.9	47.1		42.0	58.0		42.2	57.8		15.4	84.6	
Widowed/Divorced	40.5	59.5		52.8	47.2		48.3	51.7		42.1	57.9		17.7	82.3	
**Number of childrenever born**															
None	64.7	35.3	< .01	70.0	30.0	< .01	79.2	20.8	< .01	56.1	43.9	< .01	51.1	48.9	< .01
1 to 3	33.3	66.7		49.6	50.4		37.3	62.7		29.2	70.8		11.2	88.8	
4 to 7	38.1	61.9		56.5	43.5		43.9	56.1		40.6	59.4		16.7	83.3	
≥ 8	42.9	57.1		69.6	30.4		59.4	40.6		54.0	46.0		21.9	78.1	
**Religion**															
Christian-Catholic	41.8	58.2	< .01	57.0	43.0	< .01	54.5	45.5	< .01	32.9	67.1	< .01	23.3	76.7	0.58
Muslim	50.1	49.9		44.5	55.5		61.8	38.2		51.2	48.8		24.6	75.4	
Christian-Other	40.1	59.9		53.5	46.5		42.6	57.4		39.8	60.2		25.3	74.7	
Other Religion & No Religion	50.5	49.5		74.0	26.0		45.3	54.7		77.5	22.5		28.5	71.5	
**HIV stigma**															
Yes	51.8	48.2	< .01	65.7	34.3	< .01	62.1	37.9	< .01	47.9	52.1	< .01	32.0	68.0	< .01
No	35.0	65.0		46.8	53.2		41.7	58.3		32.5	67.5		18.7	81.3	
**HIV knowledge**															
Yes	46.8	53.2	< .01	49.7	50.3	< .01	41.2	58.8	< .01	35.8	64.2	< .01	19.9	80.1	< .01
No	36.1	63.9		60.6	39.4		53.6	46.4		46.5	53.5		27.2	72.8	
**Contraception**															
None	44.7	55.3	< .01	52.0	48.0	< .01	53.8	46.2	< .01	42.9	57.1	0.47	27.8	72.2	< .01
Modern	34.8	65.2		51.8	48.2		19.6	80.4		41.4	58.6		13.4	86.6	
Traditional	50.0	50.0		61.8	38.2		31.9	68.1		40.2	59.8		13.7	86.3	

Note: p < 0.01 significant.

Note: Each chi-square represents a weighted calculation by using the DHS weighted sampling unit and ‘PROC SURVEY’ command in SAS.

Table 3 shows the crude and adjusted logistic regression models for the relationship between HIV testing and contraceptive use. In the total sample and in Mozambique, women who used modern forms of contraception had higher odds of being tested for HIV compared to those who did not use contraception (adjusted odds ratio [aOR] = 1.31, 95%CI: 1.16–1.48 and aOR = 2.30, CI: 1.82–2.91, respectively). In Congo, a decrease in the odds of having an HIV test was demonstrated among those who used traditional contraception compared to those who did not use any form of contraception (aOR = 0.68, CI: 0.52–0.94). No other associations between contraception use and HIV testing were demonstrated. Sensitivity analysis excluding women who use condoms did not change the results of these models (Table 4).

**Table 3:** Logistic regression testing uptake of HIV testing by contraceptive method.

	Total	Congo	Mozambique	Nigeria	Uganda
	OR (99%C.I)	OR (99%C.I)	OR (99%C.I)	OR (99%C.I)	OR (99%C.I)
Crude					
Modern Contraception^a^	1.52 (1.36–1.69)*	1.01 (0.77–1.33)	4.77 (3.89–5.86)*	1.06 (0.87–1.30)	2.48 (1.96–3.14)*
Traditional Contraception^b^	0.81 (0.70–0.93)*	0.67 (0.51–0.89)*	2.49 (0.95–6.54)	1.12 (0.89–1.40)	2.42 (1.37–4.28)*
No Contraception	*Ref*	*Ref*	*Ref*	*Ref*	*Ref*
Adjusted ^c^^,^^d^					
Modern Contraception^a^	1.31 (1.16–1.48)*^e^	0.93 (0.68–1.27)	2.30 (1.82–2.91)*	1.00 (0.81–1.22)	1.19 (0.92–1.53)
Traditional Contraception^b^	0.92 (0.78–1.09)^e^	0.68 (0.52–0.94)*	1.46 (0.52–4.10)	0.85 (0.67–1.08)	1.19 (0.63–2.25)
No Contraception	*Ref*	*Ref*	*Ref*	*Ref*	*Ref*

Notes:

*Signifies significant relationship at p<0.01.

^a)^ Modern Contraception defined as: hormonal, sterilization, or barrier contraception methods.

^b)^ Traditional Contraception defined as: periodic abstinence, withdrawal, or other country specific forms of contraception.

^c)^ Weighted N for adjusted regression: 5431 Congo; 13453 Mozambique; 7968 Nigeria; 8640 Uganda; 35492 Total.

^d)^ Adjusted model controlled for women's marital status, number of children ever born, age, HIV stigma and comprehensive knowledge of HIV, area of residence, religion, difficulty accessing a health facility, education, and wealth index.

^e)^ Model additionally adjusted for country.

**Table 4:** Logistic regression testing uptake of HIV testing by contraceptive method, excluding condoms.

	Total	Congo	Mozambique	Nigeria	Uganda
	OR (99%C.I)	OR (99%C.I)	OR (99%C.I)	OR (99%C.I)	OR (99%C.I)
Crude					
Modern Contraception^a^	2.11 (1.84–2.42)*	1.23 (0.80–1.91)	5.49 (4.28–7.03)*	1.16 (0.92–1.45)	2.52 (1.96–3.23)*
Traditional Contraception^b^	0.79 (0.69–0.90)*	0.68 (0.52–0.89)*	2.54 (0.98–6.60)	1.10 (0.89–1.37)	2.52 (1.44–4.43)*
No Contraception	*Ref*	*Ref*	*Ref*	*Ref*	*Ref*
Adjusted ^c^^,^^d^					
Modern Contraception^a^	1.23 (1.07–1.42)*^e^	0.85 (0.53–1.36)	2.20 (1.67–2.92)*	1.01 (0.81–1.26)	1.12 (0.85–1.48)
Traditional Contraception^b^	0.91 (0.77–1.09)^e^	0.73 (0.54–0.98)*	1.48 (0.55–4.04)	0.88 (0.70–1.11)	1.24 (0.67–2.32)
No Contraception	*Ref*	*Ref*	*Ref*	*Ref*	*Ref*

Notes:

*Signifies significant relationship at a = 0.01.

^a)^ Modern Contraception defined as: hormonal, sterilization, or barrier contraception methods (excluding condoms).

^b)^ Traditional Contraception defined as: periodic abstinence, withdrawal, or other country specific forms of contraception.

^c)^ Weighted N for adjusted regression: 33249 Total; 4653 Congo; 13453 Mozambique; 6503 Nigeria; 8640 Uganda.

^d)^ Adjusted model controlled for women's marital status, number of children ever born, age, HIV stigma and comprehensive knowledge of HIV, area of residence, religion, difficulty accessing a health facility, education, and wealth index.

^e)^ Model additionally adjusted for country.

## Discussion

Understanding the relationship between HIV testing and use of contraception in high HIV transmission areas, such as SSA, is essential to decreasing the burden of HIV, especially as the prevalence of modern contraceptive use increases. This study sought to add to the literature by describing the relationship between contraception method and HIV testing in SSA. In the total sample, women who used modern forms of contraception were more likely to be tested for HIV compared to those who did not use contraception. Individual country results varied. Women in Mozambique demonstrated an increase in the odds of being tested for HIV if they were using modern forms of contraception compared to those who did not use any form of contraception. This positive association was not demonstrated in Congo, Nigeria, or Uganda. Only women in Congo demonstrated a decrease in the odds of being tested for HIV if they used traditional forms of contraception compared to those who used no contraception. Previous studies have demonstrated that in SSA 27.6% of women used modern forms of contraception [10], which is very similar to the results of the overall sample in this study (26.4%). In Mozambique, women who used modern forms of contraception had an increase in the odds of being tested for HIV. However, extremely low rates of modern contraception use were demonstrated in Mozambique (12.1%) possibly because over 50% of women reported difficulty accessing a healthcare facility. This is particularly concerning as Mozambique is a country with one of the highest prevalence rates of HIV at 11% [24]. Therefore, although women who use modern forms of contraception had higher odds of HIV testing in Mozambique, a low proportion of the overall population fell into this group. Women in SSA—and especially those in Mozambique—may benefit from services that provide both education about, and access to, modern forms of contraception and HIV testing.

Many factors decrease the uptake of HIV testing in SSA, including fear of receiving a positive HIV test result and HIV-stigma [25]. With increased access to HIV testing and counseling, the 2014 United Nations Gap Report now estimates that 48% of people know their HIV status, of which 86% of HIV positive individuals are receiving antiretroviral therapy and over three in four (76%) have achieved viral suppression [24]. However, percentages of HIV testing uptake vary greatly among SSA countries. For instance, the proportion of women tested for HIV was 50% more in Mozambique than in Congo in 2011 (33.3% total population vs. 22.5%, respectively). Uganda had the highest HIV testing rates in this study. HIV testing in Uganda also remained high as gravidity increased; in Congo, Mozambique, and Nigeria, HIV testing fluctuated as gravidity increased. Overall, uptake of HIV testing in Congo, Mozambique and Nigeria was relatively low. This data did show a substantial increase in HIV testing in each country as women who had children, compared to those who were nulliparous. Of the women who had obtained an HIV test, most received it from a government run hospital or health center: Congo (84%), Mozambique (92%), Nigeria (69%), and Uganda (68%) (results not shown). It is assumed that most of these women are partaking in HIV testing as part of antenatal care.

Because some non-condom forms of modern contraception require a clinical appointment for use, contraception appointments could provide a confidential place for access to HIV testing, education and treatment. Because of the continued fear of receiving a positive HIV test result and the associated HIV-stigma [26], providing both testing and counseling at family planning clinics may be ideal. Use of traditional forms of contraception was relatively high in Congo and Nigeria (41.8% and 22.5%, respectively). Previous studies have demonstrated that women often rely on traditional methods of birth control or abortion due to lack of knowledge about modern forms of contraception, fear of infertility, access barriers, lack of control in fertility decision making, and the association of birth control methods—especially condom use with promiscuity [27–29]. Finding ways to decrease the barriers associated with utilizing modern forms of contraception remain imperative to decreasing HIV.

### Strengths and limitations

Most previous research on this topic has assessed the effects of HIV-testing on subsequent use of contraception, particularly condoms; however, this study compared the type of contraception used (modern and traditional) and the uptake of HIV-testing. A sensitivity analysis was also conducted to explore the relationship that non-condom forms of contraception have on HIV testing. By doing this, we can gain insight to the beliefs associated with HIV risk and contraception use. This paper is not without limitations. Although we found an association between contraceptive-use by type and HIV-testing, we cannot infer a causal relationship between contraceptive use and HIV-testing. To be precise, this present study cannot depict that using a certain form of contraceptive always results in HIV-testing. In addition, this analysis used data collected across a span of two years, from 2011 to 2013. Therefore, this study acknowledges the limited ability to conduct cross-country comparisons across different time periods.

### Implications for Policy

As modern forms of contraception are introduced, healthcare providers—as well as their patients—need to be provided with fact based resources that explain what is known about the relationship between HIV acquisition and modern forms of contraception. Because the relationship between contraception utilization and subsequent HIV infection remains unclear, healthcare providers should continue to emphasize using condoms to prevent HIV acquisition. Integrating HIV education and testing into medical appointments that provide access to contraception may increase uptake of HIV testing in women who would otherwise not attend a clinic for testing. Because this study did not demonstrate a homogenous association between types of contraception and HIV testing between individual countries, there appears to be many missed opportunities for women to gain access to HIV testing. Also, providing modern forms of contraception to women in all areas of SSA could reduce vertical transmission of HIV, the number one reason for pediatric HIV infection, as contraception provides a woman the ability to plan for a future family. In Congo, women most commonly first received their current contraception from a friend or relative (42%), and in Mozambique from a health center (54%). In Nigeria and Uganda women most frequently gained access to contraceptives via the pharmacy (33%) and a private hospital or clinic (35%), respectively (results not shown). Because contraception use is often closely tied to a woman’s financial resources and education—with wealthier and more educated women displaying higher rates of contraception use—providing contraception free of charge or at a low-cost is more likely to promote use among those with the greatest unmet need [30, 31].

### Future Directions

Very little information has been published on the relationship between a woman’s method of contraception and their uptake of HIV-testing. Although condom use is the only form of contraception shown to reduce HIV transmission, inje contraception is currently the most prevalent form of contraception across SSA. Furthermore, the unclear relationship between hormonal contraceptives and HIV infection mean that women using modern contraception—but not condoms—may be at higher risk of HIV-transmission. Future studies that explore the relationship between modern—non-condom—forms of contraption and HIV are needed. Furthermore, studies that assess women’s perceptions of HIV risk while using modern forms of contraception are needed to understand if women utilizing modern forms of contraception understand their HIV risk and need for HIV testing.

## Conclusions

In this study, our pooled results demonstrated that women who used modern forms of contraception were more likely to be tested for HIV compared to those who did not use contraception. This result was true both with and without condom users in the sample. Given the increasing popularity of hormonal contraception methods, efforts should be directed at educating women about the importance of continuing to use condoms for HIV-prevention. Continued community education about HIV and the importance of knowing one’s status is also needed.
